# Eco-friendly synthesis of zinc oxide nanoparticles *via Pisum sativum* seeds and their application in the adsorptive removal of Congo red dye

**DOI:** 10.1039/d6ra02785g

**Published:** 2026-06-01

**Authors:** Amin K. Qasim, Sameera Sh. Mohammed Ameen, Adar Jamal Faris, Khalid M. Omer

**Affiliations:** a Department of Chemistry, College of Science, University of Zakho Zakho 42002 Kurdistan Region Iraq amin.qasim@uoz.edu.krd; b Department of Chemistry, College of Science, University of Sulaimani Kurdistan Region Iraq

## Abstract

This study reports the eco-friendly synthesis of zinc oxide nanoparticles (ZnO NPs) using *Pisum sativum* seed extract as a natural reducing and stabilizing agent. The biosynthesized ZnO NPs were characterized by UV-vis spectroscopy, XRD, FTIR, and FE-SEM, revealing predominantly spherical nanoparticles with uniform distribution, sizes of 30–50 nm, and aggregation driven by polarity and electrostatic interactions. Their adsorption performance was evaluated for the removal of Congo Red (CR) dye from aqueous solutions. Batch experiments demonstrated a maximum removal efficiency of ∼91% under optimal conditions (14 mg L^−1^ dye concentration, pH 6, 35 °C, 0.015 g adsorbent dose, and 40 min contact time). Adsorption followed pseudo-second-order kinetics, indicating chemisorption, and fit both Langmuir and Freundlich isotherm models, suggesting a combination of monolayer and multilayer adsorption on a heterogeneous surface. Thermodynamic analysis confirmed the process to be spontaneous, endothermic, and associated with decreased randomness at the solid–solution interface, with enhanced efficiency at higher temperatures. These results highlight the potential of *Pisum sativum*-mediated ZnO NPs as an efficient and promising eco-friendly adsorbent for wastewater treatment and dye removal. Further studies on desorption and regeneration cycles are required to evaluate long-term adsorbent sustainability.

## Abstract 2

Schematic diagram of green synthesis of ZnO NPs for adsorption of CR dye.

## Graphical abstract

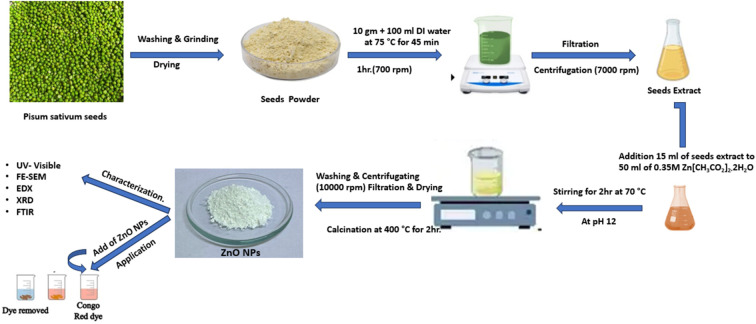

## 1 Introduction

Nanoscale science and engineering have enabled the development of nanomaterials with unique physicochemical properties that differ from bulk materials. The preparation of these nanomaterials through physical, chemical, and green synthesis methods has gained significant attention due to their wide range of applications. Their high surface area and enhanced surface reactivity make them highly effective in fields such as biological research, environmental remediation, and catalysis. These properties underscore the importance of developing efficient and sustainable methods for nanomaterial synthesis.^1,2^ The active surface area of nanoparticles (NPs) for practical applications can be modified using chemical and biological agents. While the intrinsic properties of metal nanoparticles are inherently stable, their surface characteristics, along with size and shape, can be tailored to optimize performance in environmental and biological applications.^3,4^

There is a significant need for efficient, cost-effective solutions to eliminate hazardous inorganic and organic contaminants for water recycling. Green technology is developing as an effective technique for the treatment of water waste materials.^5^

The majority of NPs are produced *via* top-down methods, including sputtering, lithography, and ball milling, as well as bottom-up techniques such as hydrothermal synthesis, spray pyrolysis, chemical vapor deposition, and sol–gel procedures. In particular, using chemical approaches, the dimensions and morphology of NPs are regulated by altering chemical concentrations and reaction parameters. However, these processes are poisonous, costly, and possibly harmful, resulting in the production of undesirable by-products. To address these challenges, green synthesis methods are garnering significant interest because to their cost-effectiveness, biocompatibility, and environmental sustainability.^6^

Certain metal NPs, including Au, Ag, CuO, ZnO, and TiO_2_, have been investigated for applications across many fields owing to their ease of preparation, safety for health, and cost-effectiveness. Zinc oxide (ZnO) is of significant commercial and industrial significance due to its distinctive characteristics and extensive applicability among different metal oxides.^7–9^

*Pisum sativum,* referred to as the garden pea, is a herbaceous annual plant belonging to the family *Fabaceae*. It is cultivated globally for its consumable seeds. The pea plant generates pods that include many seeds (peas), which may have green or yellow cotyledons at maturation. These immature peas are used as a vegetable, whether fresh, frozen, or tinned. Moreover, certain cultivars of field peas are cultivated to yield dry peas, such as the split pea, which is extracted from a fully formed pod. Peas have been produced for millennia and are a cool-season crop planted in many regions throughout.^10^

Reports indicate that *Pisum sativum* leaves and seeds include protein, carbohydrate, dietary fiber, and polyphenols. The secondary metabolites in *Pisum sativum* seeds may serve as reducing and stabilizing agents that facilitate the synthesis of Ag-doped ZnO NPs from precursor metallic salts.^11^

Dyes are very hazardous, exhibiting mutagenic and carcinogenic properties as a result of industrial organic pollution. The procedure must be efficient and cost-effective for the removal of organic contaminants from urban water. Adsorption-based methods are extensively acknowledged in several dye treatment processes. Recent research in wastewater treatment has focused on sustainable adsorbent materials that emphasize quick adsorption rates, cost-effectiveness, and enhanced adsorptive performance. Adsorption is the predominant approach for dye removal due to its operational ease and scalability.^12^

Congo Red is utilized not only in the textile and paper industries but also extensively in higher education laboratories and research institutions as a model dye for studies on adsorption, catalysis, and wastewater treatment. In Iraq, similar to numerous developing nations, laboratory effluents are frequently released into municipal or aquatic environments without adequate treatment, which may lead to localized contamination. The persistence, high solubility, and potential toxicity of Congo Red to aquatic organisms render it an appropriate and environmentally significant model pollutant for assessing the efficacy of green-synthesized adsorbents.^13,14^

The present study aims to develop a green and sustainable approach for the synthesis of zinc oxide nanoparticles (ZnO NPs) using *Pisum sativum* seed extract as a natural reducing and stabilizing agent. This eco-friendly synthesis route avoids the use of toxic chemicals and offers a cost-effective alternative for nanoparticle production. The synthesized ZnO NPs were thoroughly characterized and systematically evaluated for their adsorption performance toward the removal of Congo Red dye (CR) from aqueous solutions. Particular emphasis was placed on assessing their adsorption efficiency and potential applicability in wastewater treatment processes. The findings highlight the promise of biosynthesized ZnO NPs as an effective and environmentally benign adsorbent for the remediation of dye-contaminated water.

## 2 Experimental section

### 2.1 Materials

Zinc nitrate hexahydrate (Zn(NO_3_)_2_·6H_2_O), hydrochloric acid [HCl] and sodium-hydroxide [NaOH] have been purchased from Sigma-Aldrich, Germany. Fresh Pea (*Pisum sativum)* seeds used in this study were obtained from a local market in Duhok Province, Kurdistan Region, Iraq. All reagents used were of excellent analytical quality and used without further purification. For both experiments, deionized (DI) water was used to prepare solutions.

### 2.2 Phytochemical screening

The *Pisum sativum* seeds extract was performed for testing the biomolecules presented in seeds extract, according to the following standards methods.^15,16^

### 2.3 Preparation of the extract from *Pisum sativum* seeds

The collected fresh pea (*Pisum sativum*) seeds were thoroughly washed with DI water and dried for 15 days at room temperature and then crushed into fine powder by electrical blender, and then about 10 g were soaked in a 200 mL container flask which contained 100 mL DI water. The solution was heated at 75 °C for 45 min. The extract was cooled to room temperature, and filtered by using filter paper Whatman filter paper grade No. 1 size 110 mm to remove the remaining clusters, in addition utilizing centrifuged at 7000 rpm for 15 min to obtain a pure aqueous extract. Later the pure filtrate and kept in refrigerator at 4 °C in order to be used for further experiments.

### 2.4 Synthesis of ZnO NPs

**Fig. 1:**
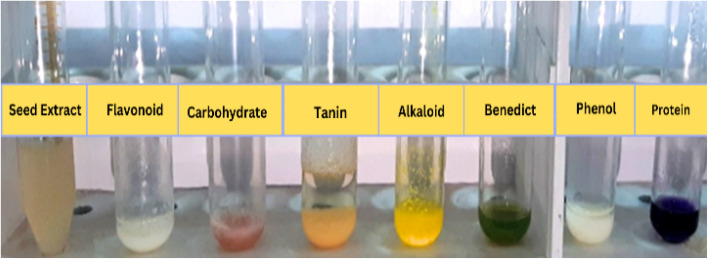
The phytochemical screening test results for *Pisum sativum* seed extract.

The bio-fabrication of ZnO NPs began with the addition of 50 mL of a 0.35 M aqueous zinc nitrate solution in a 250 mL beaker, followed by continuous stirring at 50 °C for 30 minutes. 15 mL of aqueous extract from pea (*Pisum sativum*) seeds was introduced into the zinc nitrate solution while maintaining constant stirring at the temperature indicated in Fig. 1, Step I. The reaction mixture was stirred continuously for 2 h to facilitate the electrostatic interaction between Zn^2+^ ions and the biomolecules in the aqueous seed extract, resulting in a light-yellow coloration as the phytochemicals of the extract encapsulated the Zn^2+^ ions and initiated the nucleation of ZnO NPs. At this stage, phytochemicals acted primarily as complexing and stabilizing agents for Zn^2+^ ions under acidic conditions (pH ≈ 2.0), rather than promoting ZnO nucleation directly.

In Step II, the pH of the reaction mixture was gradually adjusted to 12 by dropwise addition of freshly prepared 1.0 M NaOH under continuous stirring at 70 °C. Under alkaline conditions, the increased OH^−^ concentration facilitated the formation of Zn(OH)_2_ intermediates, followed by their conversion into ZnO nanoparticles, leading to the formation of a light-yellowish precipitate after 2 h of stirring (Fig. 1). This indicates that ZnO nucleation and growth were favored under strongly alkaline conditions, while phytochemical constituents contributed to nanoparticle stabilization.

In the concluding Step III, as seen in Fig. 1, the synthesized material was placed in an oven for 24 h at 80 °C to achieve full desiccation. A muffle furnace was used for the annealing of the dried ZnO NPs for 2 h at 400 °C. A high-quality, light cream-colored powder of ZnO NPs was used for further investigations and surface characterization. Fig. 1 illustrates the comprehensive schematic depiction of the bio-fabricated ZnO NPs.^17^

### 2.5 Instrumentation and characterization

**Scheme 1:**
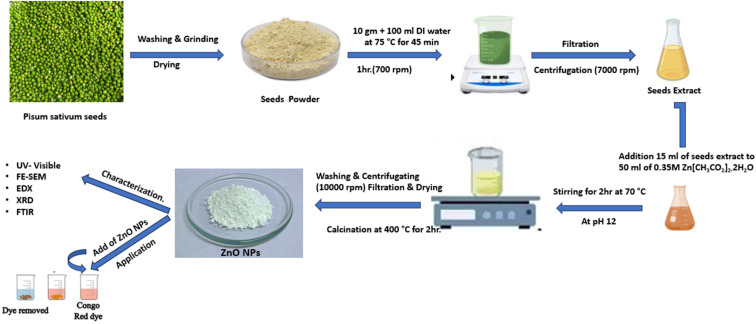
Schematic diagram of green synthesis of ZnO NPs for adsorption of CR dye.

Field emission scanning electron microscopy (FE-SEM, ZEISS, SIGMA VP, Germany) was used to study the morphology of the created microspheres. The crystallinity was assessed *via* powder X-ray diffraction (PXRD) using a D8-advanced diffractometer (Haoyuan Instrument Co Ltd, DX-2700BH) with a scan range of 2*θ* 5–70°. For elemental analysis, energy-dispersive X-ray spectrometry (EDS) mapping was used (Quanta 4500, Germany). Fourier transform infrared (FTIR) spectra were obtained using a Spectrum Two PerkinElmer in the range from 450 to 4000 cm^−1^. Absorbance spectra were acquired using a Lambda 25 spectrophotometer (PerkinElmer, USA) with a 1 cm path length cell (Scheme 1).

### 2.6 Batch adsorption experiment

A comparison study was conducted to assess the efficacy of manufactured NPs in eliminating CR dye from aqueous solutions. A batch experiment was conducted to investigate the impact of various variable conditions on the examined system, with each factor tested individually under controlled circumstances to ascertain its specific effect. The parameters included an initial dye concentration range of 2 to 14 mg. L^−1^, pH levels of 2, 4, 6, 8, and 10, contact times ranging from 0 to 40 minutes, temperatures of 20, 25, 30, 35, 40, 45, 50, and 55 °C, and the absorbance was measured and analyzed for the supernatant. The adsorption capacity, *q*_e_, was then determined using the following formula.^17^1
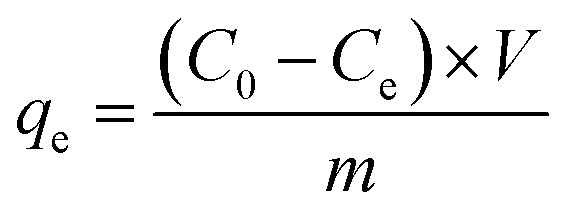
where: *q*_e_ = adsorption capacity, *C*_0_ = initial concentration, *C*_e_ = equilibrium concentration, *V* = solution volume in Liter, *m* = adsorbent mass in grams.

The adsorption efficiency can be quantified by determining the percentage removal of dye as follows:^18,19^2
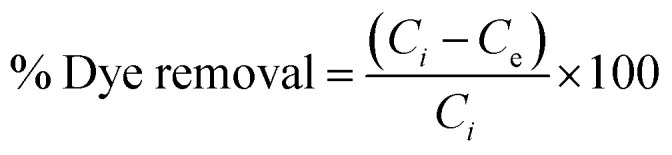
where: *C*_*i*_ = initial concentration, *C*_e_ = equilibrium concentration, adsorption isotherms.

The isotherm model of Freundlich is an empirical equation utilized for heterogeneous surfaces or surfaces with varying affinities. The logarithmic representation of the isotherm model of Freundlich is articulated as:^20^3
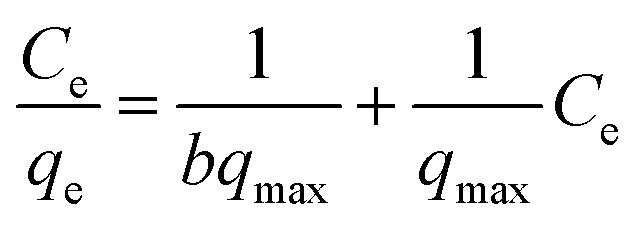
where: *C*_e_ = equilibrium concentration of dyes solution, *q*_e_ = adsorbate solid-phase concentration at equilibrium, *q*_max_ = adsorption capacity at maximum, *b* = Langmuir constant.

• The intercept value of 1/*bq*_max_ is determined with the graph of *C*_e_/*q*_e_*vs. C*_e_. *R*_L_ the dimensionless factor, often known as the separation factor, is an additional distinguishing parameter of the Langmuir isotherm, as articulated in the equation.^15^4
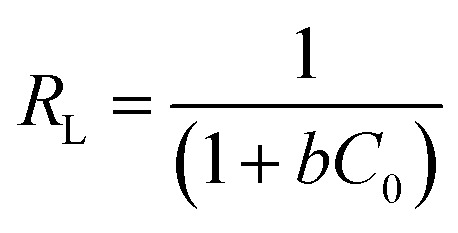
where: *R*_L_ = dimensionless factor, *C*_0_ = maximum dye concentration, *b* = Langmuir constant.

The value of RL is contingent upon the isotherm type and may indicate adverse conditions (*R*_L_ > 1), linear (*R*_L_ = 1), favorable (0 < *R*_L_ < 1), or irreversible (*R*_L_ = 0) adsorption.^21^ The Freundlich isotherm is being an empirical expression which describe the sorption process occurred on heterogeneous surfaces, or on those surfaces that they have different affinity sites. The logarithmic form for the Freundlich equation can be written as follow:5
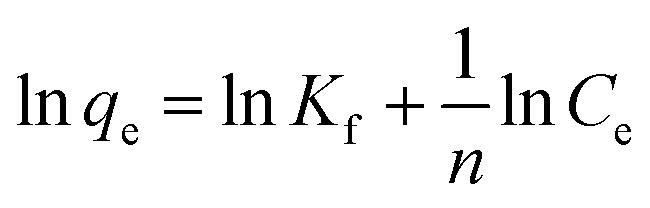
where: *K*_f_ = the adsorption capacity, *n* = intensity, *K*_f_ is a constant that represents the efficiency of adsorption. The slope of 1/*n*, ranging from 0 to 1, serves as an indicator of adsorption intensity or surface heterogeneity, with increased heterogeneity observed as the slope approaches zero. A ratio of 1/*n* less than 1 indicates a conventional Langmuir isotherm, while a value of 1/*n* greater than 1 signifies cooperative adsorption. *K*_f_ and *n* can be calculated from plotting ln *q*_e_*vs.* ln *C*_e_. The kinetics of adsorption were examined using pseudo first order and pseudo second order models. Pseudo first order is expressed by the Lagergren equation, which calculates the adsorption rate constant (*k*_1_) from the graph of ln(*q*_e_ − *q*_*t*_) *versus t*, where *q*_e_ and *q*_*t*_ denote the adsorbed amount at equilibrium and at time *t*, respectively.^21^

6 ln(*q*_e_ − *q*_*t*_) = ln *q*_e_ − *k*_1_*t*

where: *q*_e_ = adsorbed amount at equilibrium, *q*_*t*_ = adsorbed amount at time *t* Adsorption kinetics.

The (pseudo second-order) model, conversely, determines the second order rate constant (*k*_2_) from the linear graph of *t*/*q*_*t*_*versus t*.7
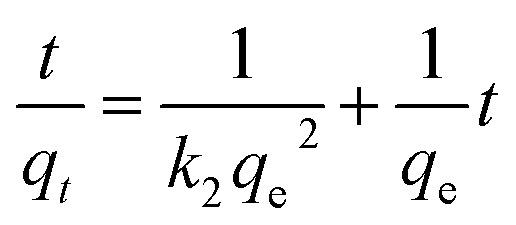


The (Δ*G*°), (Δ*H*°), (Δ*S*°) thermodynamic parameters, were calculated using the following equations that incorporate the equilibrium constant (*K*_c_), absolute temperature (*T*), and the universal gas constant (*R*). The Δ*H*° value and Δ*S*° value can be calculated from the Van't Hoff equation, the parameters were derived using the ln *K*_c_ against 1/*T* plot, where the slope indicates −Δ*H*°/*R* and the intercept signifies Δ*S*°/*R*.^22^8
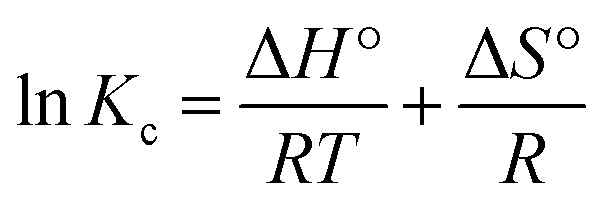

9 Δ*G*° = −*RT* ln *K*_c_

10
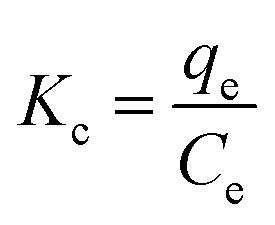


## 3 Results and discussion

### 3.1 Phytochemical screening of *Pisum sativum* seeds extract

*Pisum sativum* is believed to have originated in the Near East (Fertile Crescent region) and is now cultivated globally across temperate regions, including Europe, Asia, North America, and parts of Africa. The presence of flavonoids, alkaloid, glycoside and phenolic compounds in the cowpea extract suggests their active role in nanoparticle synthesis. These bioactive molecules are known to facilitate the reduction of metal ions to NPs and simultaneously act as capping and stabilizing agents, influencing the size, shape, and dispersion stability of the synthesized ZnO NPs. The results of phytochemical screening of *Pisum sativum* seeds extract are shown in Fig. 1 and Table 1.

**Table 1:** Phytochemical analysis of *Pisum sativum* seed extract

Active constituent	Chemical test	Results	Indication	Test ref.
Flavonoid	Lead acetate	+ve	Yellow ppt	23
Carbohydrate	Molish	+ve	Violet ring	24
Glycoside	Benedict	+ve	Red ppt	25
Tannin	Braymer	−ve	No change	26
Alkaloid	Hager	++ve	Creamy white ppt	27
Proteins & A. acid	Ninhydrin	+ve	Purple colour	28
Saponin	Foam	++ve	Foam formation	29
Phenolic	Lead acetate	++ve	White ppt	30

### 3.2 Characterization of the ZnO NPs

**Fig. 2:**
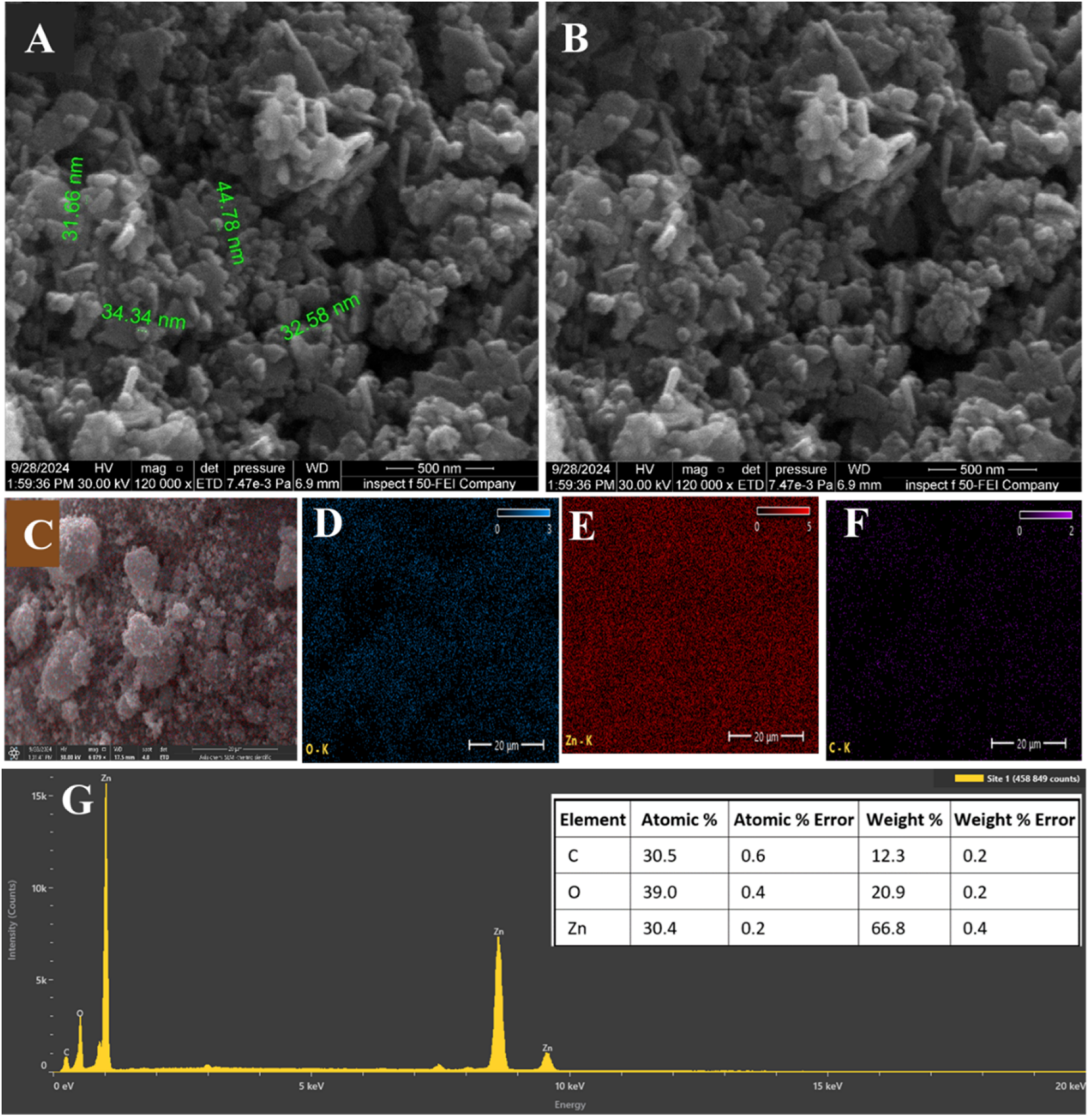
(A and B) FE-SEM of ZnO NPs. (C–F) EDX mapping of ZnO NPs. (G) EDX spectrum of ZnO NPs.

As demonstrated in Fig. 2A and B which reveals the morphological characteristics of synthesized ZnO NPs have been assessed utilizing FE-SEM. The nanoparticles have irregularly agglomerate spherical shape grains and small flakes. However, the aggregation and agglomeration of particles is likely driven by the polarity and electrostatic interactions among ZnO NPs, which promote their assembly into a more stable clustered structure. In addition, such aggregation, commonly seen in green-synthesized ZnO NPs, mainly results from van der Waals interactions and hydrogen bonding between the phytochemicals and the NPs during formation.^31^ Furthermore, as shown in Fig. 2A and B, the FE-SEM image of the green-synthesized ZnO NPs indicate that their sizes of NPs within the range of 30–50 nm. FESEM analysis revealed aggregated ZnO nanoparticles with irregular morphology and a relatively broad size distribution.

As shown in the Fig. 2G, the EDX investigation was conducted for the ZnO NPs to calculate the percentages and distribution of each element in the sample. The EDX spectrum shows the main peaks corresponding to Zn and O, confirming the successful green synthesis of ZnO NPs. The presence of additional peaks, such as carbon, may be due to minor contamination during the synthesis process; more likely, however, the carbon originates from biomolecules naturally present in the *Pisum sativum* seed extract used in the green synthesis absorbs on the surface of the ZnO NPs.

**Fig. 3:**
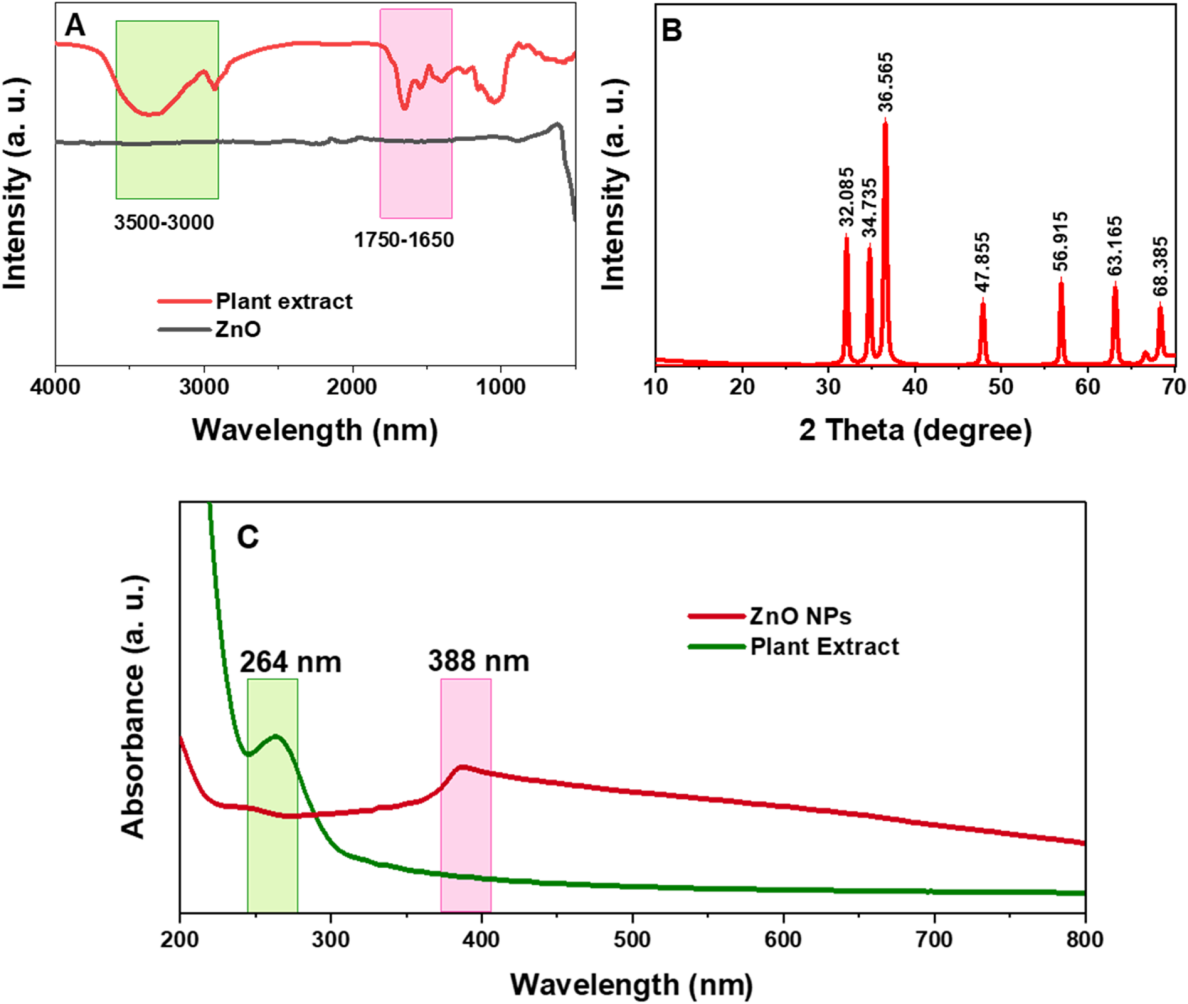
(A) FTIR of plant extract and ZnO NPs. (B) XRD of ZnO NPs. (C) UV-visible spectrum of the plant extract and ZnO NPs.

The essential functional group that responsible for reduction, cupping and stabilizing of nanoparticle identified *via* FTIR spectroscopy. The *Pisum sativum* seeds extract spectrum exhibits a wide absorption band at 1650–1750 it considers unique peak with C=O stretching in carbonyl-containing biomolecules such as tannin, flavonoids or proteins as shown in Fig. 3A, in addition at 3000–3500 cm^−1^, attributed to O–H stretching vibrations in phenolic hydroxyl groups. In the ZnO NPs, the FTIR spectra showed changes in the intensity of O–H and C=O bands, suggesting the possible involvement of phytochemical functional groups in interaction with Zn^2+^ ions and stabilization of the synthesized nanoparticles. In addition, FTIR peak intensity changes alone usually suggest possible interaction or involvement of functional groups, but they do not directly prove reduction or surface binding. In addition, the observed FTIR peak shifts between *Pisum sativum* seed extract and synthesized ZnO nanoparticles indicate the involvement of biomolecules containing hydroxyl (–OH), amine (–NH), carbonyl (C=O), and C–O functional groups. These groups likely participate in the reduction of zinc precursor ions and act as capping/stabilizing agents, preventing excessive nanoparticle growth and aggregation.

Fig. 3B shown the Crystallographic features of the X-ray diffraction (XRD) patterns for synthesized ZnO NPs. As reveals in the Fig. 3B many significant reflections peaks display at 2*θ* value of 31.9°, 34,5°, 36.4°, 48°, 56.9°, 63.1°, 66.4°, 67.1° in line with a hexagonal wurtzite structure (JCPDS 36-1451),^32^ corresponding to (100), (002), (101), (102), (110), (103), (200) and (112) planes, respectively. The absence of any interfering peaks demonstrates the purity of the synthesized ZnO NPs. Furthermore, the average crystallite size of the synthesized ZnO NPs was estimated using the Scherrer equation to be 35 nm. The strong and sharp peaks demonstrate that the material is extremely crystalline.

The UV-vis absorption spectra have been used to exam the optical characteristics of ZnO NPs, for both the plant extract and ZnO NPs the UV-vis spectra between 200 and 800 nm. The plant extract demonstrations a specific absorption maximum at 264 nm, for the reason π → π* electronic transitions in conjugated aromatic systems typical of phenolic chemicals and flavonoids in this area as shown in the Fig. 3C. Furthermore, as an indication of successful nanoparticle synthesis, ZnO NPs exhibit a distinct red-shifted absorption peak at 388 nm in their UV-vis spectra. This peak is clearly separated from the absorption features of the plant extract, confirming the formation of ZnO nanoparticles and reflecting their characteristic optical properties, which arise from their nanoscale size and surface interactions.

### 3.3 Adsorption study

#### 3.3.1 Effect of initial concentration

**Fig. 4:**
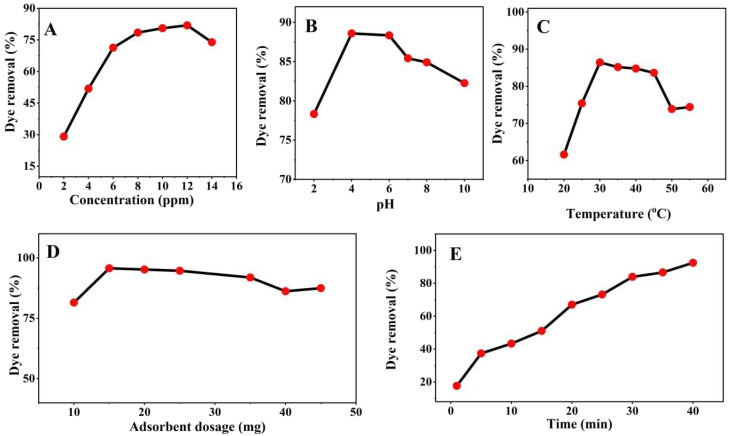
Effect of five important parameters on the adsorption capacity and dye removal efficiency of CR onto ZnO NPs. (A) Effect of concentration on adsorption capacity. (B) Effect of pH on dye removal efficiency. (C) Effect of temperature on adsorption capacity. (D) Effect of adsorbent dosage on dye removal efficiency. (E) Effect of contact time on adsorption.

Investigated the CR dye concentration effect on the adsorption process with various concentration of 2, 4, 6, 8, 10, 12, and 14 mg L^−1^, while the other parameters keep constant including (80 min) time, (25 °C) temperature with pH (7.0), and adsorbent dose (0.01 g). As shown in Fig. 4A the removal percentage increased with increasing initial dye concentration, and the maximum removal efficiency (71.62%) was achieved at 14 mg L^−1^. The increase in removal efficiency at higher dye concentrations may be attributed to the greater availability of dye molecules, which enhances their interaction with the active adsorption sites on the ZnO nanoparticles until near-saturation conditions are approached.

#### 3.3.2 Effect of pH

ZnO NPs was utilized for adsorption of CR dye concentration and the effluence of pH on the adsorption was investigated to select the optimal pH level. CR solution was prepared with different solution and different pH level from 2.0 to 10, but others parameters remain constant like (dye concentration of 14 mg L^−1^, duration of 80 min, adsorbent dosage of 0.01 g, and ambient temperature of 25 °C). As shown in the Fig. 4B the increase CR dye removal with increasing pH up to 6 with removal efficiency 85.496% by ZnO NPs and the adsorption capacity subsequently declined when the pH was raised beyond 6 up to 10. Finally selected pH 6 as optimum for adsorption CR dye and elucidate the potential adsorption mechanism.

The pH-dependent adsorption behavior can be explained based on the point of zero charge (pH_p_zc) of ZnO nanoparticles. Previous studies reported pH_p_zc values of biosynthesized ZnO nanoparticles in the range of 6.71–7.14 for Congo Red adsorption systems. At pH values below pH_p_zc, the ZnO nanoparticle surface becomes positively charged, which promotes electrostatic attraction toward the anionic Congo Red molecules. Therefore, the maximum adsorption observed at pH 6 in the present study is consistent with literature findings. At higher pH values, surface charge reversal and increased OH^−^ competition reduce adsorption efficiency.^30,33,34^

#### 3.3.3 Effect of temperature

The temperature is one of important variable during adsorption of CR dye *via* ZnO NPs, therefor various temperature has been investigated from (15 to 60 °C). The other parameters were kept constant, including dye concentration of 14 mg L^−1^, duration of 80 min, adsorbent dosage of 0.01 g, pH 6 and removal percentage reached maximum at temperature of 35 °C as shown in the Fig. 4C due to stronger physical adsorption and favor able adsorption interaction; this temperature is selected as the best setting for the adsorption process. This improvement at moderate temperature can be attributed to enhanced diffusion of dye molecules and favorable interaction with the active sites of ZnO nanoparticles, promoting physical adsorption. However, at temperatures above 35 °C, the removal efficiency decreased, which may be due to the weakening of adsorption forces, partial desorption of dye molecules, and possible aggregation of nanoparticles that reduces the available active surface area. Based on these results, 35 °C was selected as the optimum temperature for the adsorption process.

#### 3.3.4 Effect of CP NPs dosage

Different amount of ZnO nanoparticle dose including 0.010 to 0.15 gm per 10 mL of CR dye has been investigated while another variable is optimized and remains constant dye concentration of 14 mg L^−1^, duration of 80 min, maximum temperature is 35 °C and pH 6. Fig. 4D illustrates that the maximum dye removal efficiency was 93.026% for ZnO NPs at a dosage NPs as a catalyst of 0.015 g. This is attributable to the improved convenience of the adsorbent sites at higher doses of the adsorbent. Elsewhere this optimal dose, the removal capacity begins to decline slightly; the best description is agglomeration prospect, which decreases the effective surface area and limits CR dye accessibility to active spots. Thus 0.015 g of the catalyst is the optimal catalytic dose, which is essential for maximizing removal capacity; this dose is selected as the best setting for the adsorption process.

#### 3.3.5 Effect of contact time

The final important parameter contact time also should be fixed after all other variables selected depend on the maximum absorbance capacity of the CR dye. The effect of time is studied from 0 to 40 min and maximum efficiency capacity is 90.916% recorded at 40 min as shown in Fig. 4E. The removal capacity increases rapidly within the first 5–30 min, due to abundant active sites on the ZnO nanoparticle surface. A slower increase in removal efficiency was observed at later contact times, suggesting gradual occupation of adsorption sites as the system approached saturation. Therefore, 40 min was selected as the optimal contact time under the investigated experimental conditions.

### 3.4 Adsorption isotherm

**Fig. 5:**
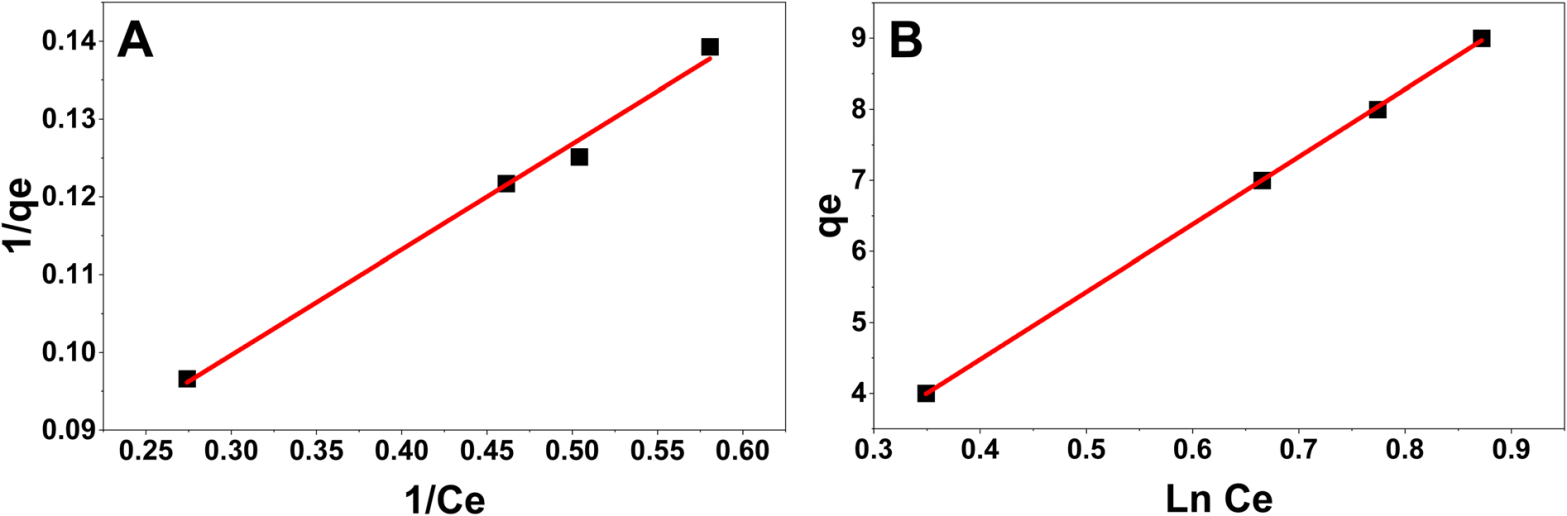
(A) Langmuir isotherm (B) Freundlich isotherm.

The Langmuir isotherm seen in Fig. 5A demonstrates strong linearity, indicating monolayer adsorption of CR molecules onto a uniform ZnO surface with limited and energetically comparable active sites. Conversely, the Freundlich isotherm shown in Fig. 5B demonstrates a commendable match, affirming the multilayer heterogeneous characteristics of the adsorbent surface and the participation of multilayer adsorption at elevated dye concentrations. Indicating the possible presence of heterogeneous adsorption sites and multilayer adsorption behavior. The applicability of both Langmuir and Freundlich models suggests that CR adsorption onto *Pisum sativum*-mediated ZnO NPs may involve a complex adsorption mechanism influenced by surface heterogeneity and adsorbent–adsorbate interactions.

### 3.5 Adsorption kinetics

**Fig. 6:**
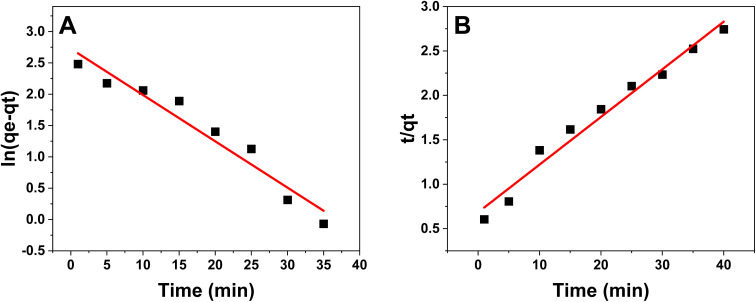
(A) Pseudo-first-order kinetic model. (B) Pseudo-second-order kinetic model.

Fig. 6A and B illustrates the use of pseudo-first order and pseudo-second order kinetic models to examine the adsorption kinetics. Both models were calibrated based on the adsorption data, and the correlation coefficient values were compared, approaching 1. The correlation coefficients for the pseudo-first-order and pseudo-second-order models were determined to be 0.9473 and 0.9749, respectively, for green produced ZnO NPs. Consequently, the pseudo-second-order model exhibits superior correlation values compared to the pseudo-first-order model, suggesting a more accurate representation of the adsorption process. The findings indicate that the adsorption behavior of CR dye on the surface of ZnO NPs adheres to a chemical adsorption mechanism, including bonding forces or the exchange of ions and electrons between the dye molecules and the ZnO NPs.

In addition, for the pseudo-first-order model, the rate constant (*k*_1_) and calculated equilibrium adsorption capacity (*q*_e_, cal) were found to be 0.0739 min^−1^ and 15.28 mg g^−1^, respectively. In contrast, the pseudo-second-order model showed a rate constant (*k*_2_) of 0.0042 g mg^−1^ min^−1^ and a calculated adsorption capacity of 18.64 mg g^−1^. The higher correlation coefficient of the pseudo-second-order model, together with the closer agreement between its calculated adsorption capacity and the experimental value (*q*_e_, exp = 18.642 804 mg g^−1^), confirms that this model more accurately describes the adsorption behavior of CR dye onto ZnO NPs.

These findings suggest that the adsorption process is mainly governed by chemisorption, involving electron sharing or exchange between CR dye molecules and active sites on the ZnO nanoparticle surface.^35^

### 3.6 Thermodynamic study

**Fig. 7:**
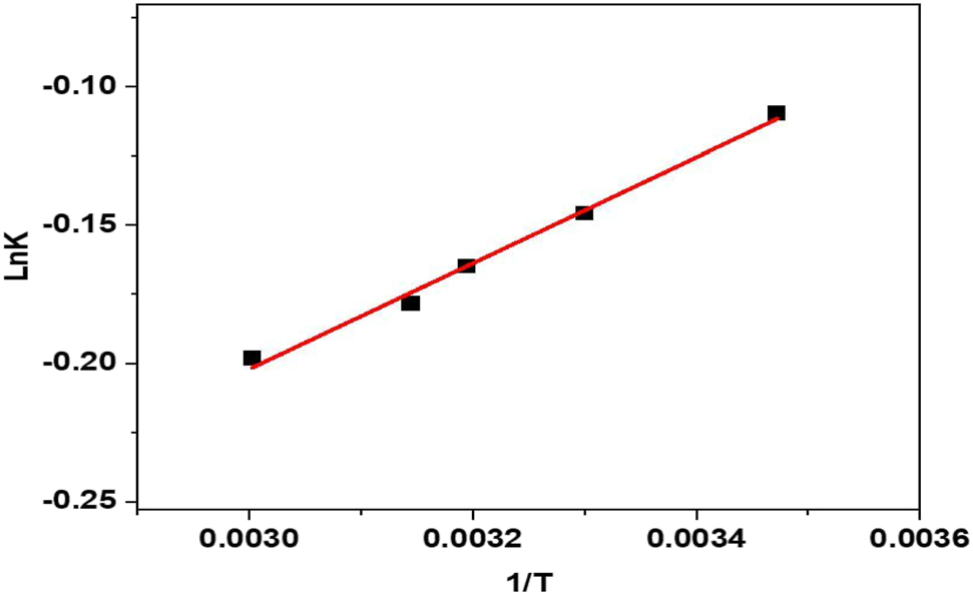
Thermodynamic study of CR adsorption onto ZnO NPs.

The thermodynamic behavior of CR adsorption onto eco-friendly synthesized ZnO NPs was assessed using the Van't Hoff plot (ln *K versus* 1/*T*), which exhibited a strong linear correlation with a high coefficient of determination (*R*^2^ = 0.993), as illustrated in Fig. 7, confirming the reliability of the thermodynamic parameters. The positive enthalpy change (Δ*H* = 0.431 kJ mol^−1^) indicates that the adsorption process is endothermic, implying that higher temperatures enhance dye absorption. The negative entropy shift (Δ*S* = −0.529 J mol^−1^ K^−1^) indicates a reduction in randomness at the solid–solution interface during adsorption, possibly resulting from the systematic attachment of CR molecules to the ZnO NPs surface. Moreover, the negative Gibbs free energy values (Δ*G*) across the examined temperature range (15–60 °C) substantiate that the adsorption process is spontaneous, as indicated in Table 2, with increasing negativity at elevated temperatures, underscoring the augmented feasibility and affinity between CR dye and ZnO NPs. The results indicate that *Pisum sativum* seed-mediated ZnO NPs have thermodynamically favorable and temperature-dependent adsorption characteristics, underscoring its potential use in the sustainable elimination of organic dyes from wastewater.

**Table 2:** Thermodynamic parameters of CR adsorption onto ZnO NPs

NPs	Δ*H* (kJ mol^−1^)	Δ*S* (J mol^−1^ K^−1^)	Δ*G* (kJ mol^−1^)										*R* ^2^
			15 °C	20 °C	25 °C	30 °C	35 °C	40 °C	45 °C	50 °C	55 °C	60 °C	
ZnO	0.431	−0.529	−17.14	−16.52	−17.31	−17.94	−18.20	−18.48	−18.74	−18.70	−19.05	−19.57	0.993

### 3.7 Mechanism of adsorption

The absorption mechanism of CR dye onto ZnO NPs utilizing *Pisum sativum* seed extract adheres to both monolayer and multilayer adsorption models. Subsequent application of isotherm models, specifically Langmuir and Freundlich, confirms that the surface of ZnO NPs possesses a range of adsorption sites with varying affinities for CR dye molecules. The chemisorption process predominates in the mechanism of CR dye adsorption onto the surface of ZnO NPs, so the kinetics order is pseudo-second.

**Fig. 8:**
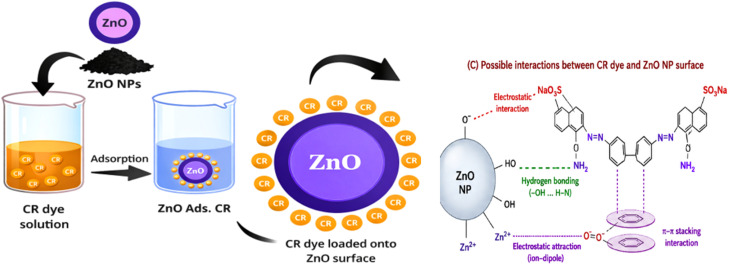
Schematic illustration of the adsorption mechanism of the CR dye onto ZnO NPs.

Fig. 8 Illustrates a schematic representation of the adsorption method by which CR dye molecules interact with ZnO NPs *via* electrostatic interactions, hydrogen bonding between dye functional groups and surface hydroxyl groups, and possible interactions with phytochemical residues present on the nanoparticle surface. Possible non-covalent interactions between CR molecules and phytochemical residues associated with the ZnO nanoparticle surface.

**Fig. 9:**
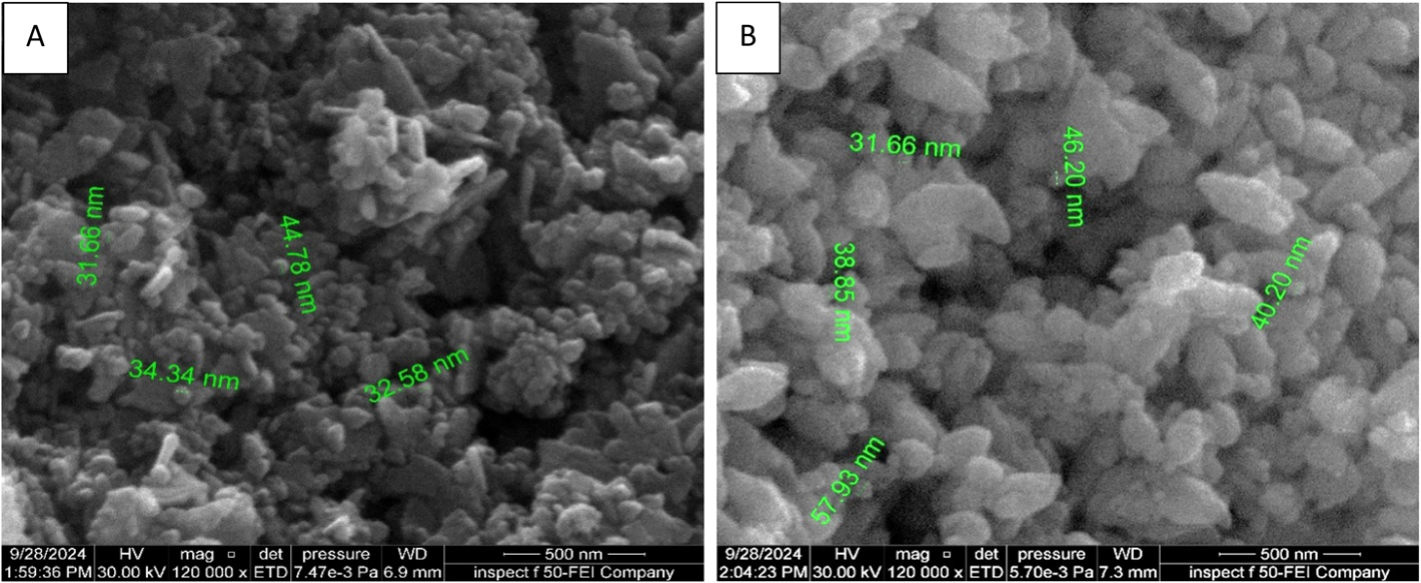
FE-SEM images of ZnO NPs (A) before adsorption and (B) after adsorption.

After adsorption, noticeable changes in nanoparticle morphology and size were observed, as shown in Fig. 9B, where particle dimensions increased to above 50 nm and aggregation became evident. These changes can be attributed to the adsorption of CR molecules onto the ZnO NP surface, forming an organic coating layer that increases the apparent particle size. Additionally, CR molecules containing multiple aromatic rings and functional groups may act as bridges between adjacent nanoparticles, promoting particle–particle interactions and aggregation. The reduction in surface charge stabilization after dye adsorption may further contribute to nanoparticle clustering and agglomeration.

Fig. 9 presents FE-SEM images of the synthesized ZnO NPs before and after CR dye adsorption. As shown in Fig. 9A, the nanoparticles are predominantly spherical, uniformly distributed, and range in size from 30 to 50 nm. After dye adsorption, noticeable changes occur in both morphology and size, with particle dimensions increasing to over 50 nm and aggregation becoming evident, as illustrated in Fig. 9B. Additionally, the adsorbed nanoparticles exhibit a slight increase in surface roughness. This comparison clearly demonstrates the effect of CR dye adsorption on the size, morphology, and distribution of ZnO nanoparticles.

## 4 Conclusions

In summary, ZnO nanoparticles were successfully synthesized *via* an eco-friendly green method using *Pisum sativum* seed extract, confirmed by a characteristic UV-vis absorption peak at 388 nm, XRD patterns consistent with a hexagonal wurtzite structure, and FTIR analysis indicating the involvement of seed biomolecules in nanoparticle reduction and stabilization. Batch adsorption studies revealed that ZnO NPs achieved a maximum CR dye removal efficiency of approximately 91% under optimal conditions (14 mg L^−1^ dye concentration, pH 6, 35 °C, 0.015 g adsorbent dose, and 40 min contact time). The adsorption process followed pseudo-second-order kinetics, indicating chemisorption as the predominant mechanism. Isotherm analyses demonstrated that both Langmuir and Freundlich models were applicable, suggesting a combination of monolayer and multilayer adsorption on a heterogeneous nanoparticle surface. Thermodynamic parameters confirmed that the adsorption is spontaneous (negative Δ*G*°), endothermic (positive Δ*H*°), and associated with decreased randomness at the solid–solution interface (negative Δ*S*°), with enhanced efficiency at elevated temperatures. These findings collectively demonstrate that *Pisum sativum*-mediated ZnO nanoparticles possess well-defined optical, structural, and morphological properties, along with high adsorption efficiency, favorable kinetics, and thermodynamically viable behavior, highlighting their potential as effective, sustainable, and environmentally friendly adsorbents for wastewater treatment and dye removal applications.

## Ethics approval

Ethical approval was not required for this study because it did not involve human participants, animals, or the use of sensitive data.

## Consent for publication

All authors have read and approved the final version of the manuscript and agreed to its publication.

## Author contributions

AJF & SSHM: conceptualization, data curation, formal analysis, investigation, methodology, resources, software, visualization, and writing – original draft.: supervision, methodology, and AKQ: writing – review and editing. KMO: supervision, validation, and writing – review and editing.

## Conflicts of interest

The authors declare that there are no conflicts of interest regarding the publication of this paper.

## Data Availability

The datasets generated and/or analyzed during the current study are available from the corresponding author upon reasonable request.
